# Risk factors and early markers for echovirus type 11 associated haemorrhage-hepatitis syndrome in neonates, a retrospective cohort study

**DOI:** 10.3389/fped.2023.1063558

**Published:** 2023-04-06

**Authors:** Ping Wang, Yi Xu, Ming Liu, Huixian Li, Hui Wang, Yumei Liu, Bin Wang, Shiwen Xia, Heng Su, Mou Wei, Li Tao, Xiaowen Chen, Bingtai Lu, Xiaoqiong Gu, Hui Lyu, Wei Zhou, Huayan Zhang, Sitang Gong

**Affiliations:** ^1^ Division of Neonatology and Center for Newborn Care, Guangzhou Women and Children's Medical Center, Guangzhou Medical University, Guangzhou, China; ^2^ Division of Infectious Disease, Guangzhou Women and Children's Medical Center, Guangzhou Medical University, Guangzhou, China; ^3^ Institute of Pediatrics, Guangzhou Women and Children's Medical Center, Guangzhou Medical University, Guangzhou, China; ^4^ Data Center, Guangdong Provincial People's Hospital, Guangzhou, China; ^5^ Division of Neonatology, Tongji Medical College, Maternal and Child Health Hospital of Hubei Province, Huazhong University of Science and Technology, Wuhan, China; ^6^ Division of Neonatology, Guangdong Provincial People's Hospital, Guangzhou, China; ^7^ Division of Neonatology, Zhujiang Hospital of Southern Medical University, Guangzhou, China; ^8^ Medical Research Center of Guangdong Provincial People's Hospital, Guangdong Academy of Medical Sciences, Guangzhou, China; ^9^ Department of Clinical Laboratory, Guangzhou Women and Children's Medical Center, Guangzhou Medical University, Guangzhou, China; ^10^ Division of Neonatology, Children's Hospital of Philadelphia and University of Pennsylvania Perelman School of Medicine, Philadelphia, United States; ^11^ Division of Gestroenterology, Guangzhou Women and Children's Medical Center, Guangzhou Medical University, Guangzhou, China

**Keywords:** newborn, echovirus 11, intensive care medicine, hepatitis, critical infection

## Abstract

**Background:**

Echovirus type 11(E-11) can cause fatal haemorrhage-hepatitis syndrome in neonates. This study aims to investigate clinical risk factors and early markers of E-11 associated neonatal haemorrhage-hepatitis syndrome.

**Methods:**

This is a multicentre retrospective cohort study of 105 neonates with E-11 infection in China. Patients with haemorrhage-hepatitis syndrome (the severe group) were compared with those with mild disease. Clinical risk factors and early markers of haemorrhage-hepatitis syndrome were analysed. In addition, cytokine analysis were performed in selective patients to explore the immune responses.

**Results:**

In addition to prematurity, low birth weight, premature rupture of fetal membrane, total parenteral nutrition (PN) (OR, 28.7; 95% CI, 2.8–295.1) and partial PN (OR, 12.9; 95% CI, 2.2–77.5) prior to the onset of disease were identified as risk factors of developing haemorrhage-hepatitis syndrome. Progressive decrease in haemoglobin levels (per 10 g/L; OR, 1.5; 95% CI, 1.1–2.0) and platelet (PLT) < 140 × 10⁹/L at early stage of illness (OR, 17.7; 95% CI, 1.4–221.5) were associated with the development of haemorrhage-hepatitis syndrome. Immunological workup revealed significantly increased interferon-inducible protein-10(IP-10) (*P *< 0.0005) but decreased IFN-α (*P *< 0.05) in peripheral blood in severe patients compared with the mild cases.

**Conclusions:**

PN may potentiate the development of E-11 associated haemorrhage-hepatitis syndrome. Early onset of thrombocytopenia and decreased haemoglobin could be helpful in early identification of neonates with the disease. The low level of IFN-α and elevated expression of IP-10 may promote the progression of haemorrhage-hepatitis syndrome.

## Introduction

Human echovirus, an enterovirus subgroup B virus, is an important cause of perinatal infection in the neonates. Among the various subtypes of echoviruses, echovirus type 11 (E-11) is an important cause of perinatal infection in neonates, which spreads rapidly over large geographic areas (1–3). It has been identified as the causal agent of haemorrhage-hepatitis syndrome (hepatitis with liver dysfunction and coagulopathy) in neonates and of outbreaks in the neonatal intensive care units (NICUs) (4–11). Data from the United States National Enterovirus Surveillance System reported significantly higher mortality from E-11 infection in neonates as compared with infants older than 1 month (19% vs. 3.2%, respectively) (12). However, current data specific to neonatal haemorrhage-hepatitis syndrome owing to E-11 infection are limited and mostly from single case reports or small number cases series. Specific risk factors and early markers associated with haemorrhage-hepatitis syndrome in neonates and the immune response remain unknown. More data is needed to better characterize the neonatal disease and help to elucidate why some neonates are prone to haemorrhage-hepatitis syndrome.

The data from an E-11 infection outbreak in neonates in China in 2019 were collected. The main objectives of this study were as follows: (1) to summarize the clinical features of neonates with haemorrhage-hepatitis syndrome and (2) to explore immunological parameters associated with haemorrhage-hepatitis syndrome. These data may help clinicians to identify and manage severe E-11 infection in neonates in a timely manner and may bring some insights on the immunological responses in neonates with E-11 associated haemorrhage-hepatitis syndrome.

## Materials and methods

### Study design and patients

This was a multicentre retrospective cohort study. Neonates with E-11 infection who were admitted to one NICU in Hubei province and three NICUs in Guangdong province in China between April and October 2019 were included. A confirmed neonatal case was defined with laboratory evidence of E-11 infection detected by reverse transcription polymerase chain reaction (RT-PCR). Investigators at each participating hospital reviewed the medical records of the included patients and obtained the following data: (1) basic demographic data and perinatal history; (2) clinical course, treatments and outcomes; (3) laboratory and immunological testing. Complete blood counts were all obtained in the first three days of E-11 infection onset, and the other laboratory tests were obtained within the first week. Blood samples for immunological testing were taken between seven and ten days of illness.

Patients were categorised into two groups (mild and severe) based on the absence or presence of haemorrhage-hepatitis syndrome. Patients who were asymptomatic or with mild symptoms without hepatic dysfunction (elevated liver enzymes) and coagulopathy were categorised as the mild group. Patients in the severe group had hepatic dysfunction and coagulopathy, with the most severe ones having liver failure (defined as elevated liver enzymes with an international normalized ratio ≥2) and disseminated intravascular coagulation (DIC) (13). We reviewed the disease course from the onset of illness to up to 4 months after discharge. Patient characteristics were compared to identify risk factors and early markers associated with the development of haemorrhage-hepatitis syndrome.

This study was approved by the Institutional Review Board of Guangzhou Women and Children's Medical Centre [GWCMC- (2020)63201]. Written informed consent was collected from the parents of the participants before their inclusion in the study.

### Pathogen detection

E-11 infection was identified using RT-PCR (14, 15). E-11 RNA was isolated from the throat and rectal swabs, blood specimens and/or cerebrospinal fluid of the patients (16), The extracted RNA was digested and fragmented, end repaired, and followed by adaptor ligation and PCR amplification to construct the library. The Agilent 2,100 Bioanalyzer quality control library fragment size was about 300 bp, and the Qubit dsDNA HS Assay Kit (Thermo Fisher Scientific Inc.) was used. The concentration of DNA libraries is controlled, and the constructed libraries are pooled in equal masses according to the detected concentration. The pooled libraries are looped to form a single-stranded loop structure. After rolling circle replication (RCA), DNB nanospheres are generated. The prepared DNB nanospheres are loaded onto the sequencing chip, and BGISEQ-50/MGISEQ-2000 is used for sequencing. The whole-genome sequence was compared and analysed in the National Centre of Biotechnology Information (NCBI) database.

### Cytokine measurement and lymphocyte subset immune-phenotyping

A total of 13 cytokines and lymphocyte subsets were analysed in plasma samples obtained from six patients in one NICU in Guangdong and were compared to age-matched neonatal controls that had no symptoms of any infection. The tested cytokines included interleukin (IL)-1β, IL-6, tumour necrosis factor-alpha (TNF-α), interferon (IFN)-inducible protein-10 (IP-10), IFN-*λ*1, IL-8, IL-12p70, IFN-α2, IFN-*λ*2/3, granulocyte-macrophage colony-stimulating factor (GM-CSF), IFN-*β*, IL-10 and IFN-*γ*). Two blood samples were collected from E-11 infected infants between seven and ten days of their illness and plasma were stored at −80°C. The protein levels of cytokines were tested. For each patient, the time point with the highest inflammatory cytokine levels was considered an acute inflammatory state and thus selected for presentation.

Cytokine levels in plasma were measured using LEGENDplex™ Human Anti-Virus Response Panel (Biolegend, Cat#740118) according to the manufacturer's instructions. Samples were read by flow cytometry. LEGENDplex v8.0 was used to analyze the results. For results under the limits of detection (LOD), the lowest value of the standard for each of the cytokines was used for statistical analysis.

Peripheral blood mononuclear cells (PBMCs) were extracted from peripheral blood of the six patients and sub-populations of PBMCs were identified by using flow cytometry (FACS Aria Sorp, BD Biosciences, San Jose, CA, USA) and analysed with Flowjo 10.4 software (FlowJo LLC, Ashland, OR, USA). Peripheral blood samples were also stimulated with Phorbol-12-myristate-13-acetate (PMA). Neutrophil ROS production was measured by flow cytometry using the Dihydroethidium, DHE dye.

All antibodies used in this study were purchased from BioLegend, San Diego, CA, USA. These included BV785 anti-human CD3 (Cat # 344842), PerCP/Cy5.5 anti-human CD235 (Cat # 349110), BV510 anti-human CD8 (Cat # 344732), BV650 anti-human CD4 (Cat # 300536), FITC anti-human CD25 (Cat # 356108), PE_Dazzle594 anti-human CD127 (Cat # 351336), BV785 anti-human CD19 (Cat # 302240), PE_Dazzle594 anti-human CD269 (Cat # 357512), PE anti-human CD38 (Cat # 303506), BV510 anti-human CD138 (Cat # 356518).

### Statistics

Standard descriptive statistics were used to summarise the demographic and clinical data. Categorical variables were expressed as counts and percentages. The Cochran-Armitage trend or Kruskal-Wallis tests were performed to determine if significant differences existed among patients in different groups. Continuous variables that did not follow a normal distribution, as determined by the Shapiro-Wilk test, were expressed as the median and interquartile range (IQR) and were compared using the Kruskal-Wallis test. Univariate logistic regression models were used to assess the risk factors for severe infection with liver dysfunction and poor outcomes. Variables with statistical significance were further analysed using a multivariate logistic regression model. A complete-case analysis was initially performed, followed by a multiple imputation sensitivity analysis. Multiple imputation sensitivity analyses based on multistage imputation were performed to assess whether our results were biased because of missing data. The multistage imputation used the Markov Chain Monte Carlo (MCMC) and monotone regression methods to achieve approximate stationarity in fewer iterations (17). Variables selected in any one of the five imputed datasets were aggregated using PROC MIANALYZE. All probability values were two-sided, and overall statistical significance was defined as *P *< 0.05. For post-hoc pairwise comparisons, the Bonferroni's method was used, and the significance levels were 0.0167 (three pairwise comparisons). Analyses were performed using SAS 9.4 Windows software (SAS Institute, Inc., Cary, NC, USA, 2015).

## Results

### Patient characteristics

A total of 105 neonates with E-11 infection were included in the study; 59 were male patients and 46 were female patients. These infants were born at a median gestational age of 38^+3^ (37–39^+1^) weeks with a median birth weight of 3100 g (2950–3285 g). E-11 infection occurred at a median age of 13 (3–20) days after birth. Of these 105 infants, 71% (75/105) of infants had mild disease, 29% (30/105) had severe disease. The case-fatality rate was 6% (6/105). Compared with the mild groups, the severe group had significantly higher percentages of infants born at <37 weeks (12% vs. 50%), with <2500 g birth weight (8% vs. 30%), and had onset of the disease at <3 days of life (21% vs. 33%) (*P *< 0.05). We found a statistically significant difference in the percentage of patients receiving parenteral nutrition (PN) prior to the onset of E-11 infection between the mild and severe groups (22% vs. 67%, respectively) (*P* < 0.001). (Table 1). The occurrence of E-11 infection peaked in June and July, followed by a decline in August in this cohort from two provinces that are approximately 1,000 km (approximately 600 miles) apart in China.

**Table 1:** Clinical characteristics of the study population.

Characteristics	All Patients (*n* = 105)	Mild (*n* = 75)	Severe (*n* = 30)	*P* value^‡^
**Demographics**				
**Gender**				
Male, *n* (%)	59 (56)	38 (51)	21 (70)	0.071
Gestational Age, median(IQR)	38^+3^ (37–39^+1^)	38^+6^ (38–39^+1^)	37^+1^ (34–39^+1^)	0.012
<37w, *n* (%)	24 (23)	9 (12)	15 (50)	<0.001
**Birthweight, median(IQR)**	3,100 (2950–3285)	3,120 (2980–3320)	2,875 (2400–3200)	0.003
<2500** **g, *n* (%)	15 (14)	6 (8)	9 (30)	0.004
**Mode of delivery**				
Vaginal delivery, *n* (%)	50 (48)	37 (49)	13 (43)	0.578
Caesarean section, *n* (%)	55 (52)	38 (51)	17 (57)	
Age at disease onset, median(IQR)	13 (3–20)	16 (6–21)	6.5 (1–14)	0.007
<3d, *n* (%)	26 (25)	16 (21)	10 (33)	0.022
3–7d, *n* (%)	16 (15)	8 (11)	8 (27)	
≥8d, *n* (%)	63 (60)	51 (68)	12 (40)	
**Feeding pattern at disease onset**				
TEN, *n* (%)	69 (66)	59 (79)	10 (33)	<0.001
PPN, *n* (%)	22 (21)	11 (15)	11 (37)	
TPN, *n* (%)	14 (13)	5 (7)	9 (30)	
Mode of feeding at disease onset				
Breast-feeding/Donated breast-feeding	64 (61)	46 (61)	18 (60)	0.947
Mixed feeding	32 (30)	23 (31)	9 (30)	
Formula feeding	9 (9)	6 (8)	3 (10)	
**Maternal History**				
Fever/diarrhea prior to delivery, *n* (%)	4 (4)	2 (3)	2 (7)	0.687^a^
Premature rupture of membrane, *n* (%)	13 (12)	6 (8)	7 (23)	0.031
**Laboratory findings**				
White blood cell count (10⁹/L), median(IQR)^#^	9.6 (7.9–11.9)/103	9.5 (7.9–11.4)/73	10.2 (8.2–12.6)/30	0.569
<5, *n* (%)	3 (3)/103	2 (3)/73	1 (3)/30	0.568
5–12, *n* (%)	76 (74)/103	56 (77)/73	20 (67)/30	
>12, *n* (%)	24 (23)/103	15 (21)/73	9 (30)/30	
Lymphocyte count (10⁹/L), median(IQR)^#^	3.3 (2.4–4.3)/101	3.2 (2.4–4.2)/73	4.1 (2.6–5.3)/28	0.157
<1.5, *n* (%)	10 (10)/101	6 (8)/73	4 (14)/28	0.361
Platelet count (10⁹/L), median (IQR)^#^	185 (172–196)/103	186 (179–196)/73	120 (63–178)/30	<0.001
<140, *n*(%)	16 (16)/103	1 (1)/73	15 (50)/30	<0.001
Alanine aminotransferase (U/L), median(IQR)^#^	20 (12–25)/101	17.9 (11.5–23)/72	29 (17–64.2)/29	0.001^a^
>50, *n* (%)	9 (9)/101	0 (0)/72	9 (31)/29	<0.001
Aspartate aminotransferase (U/L), median(IQR)^#^	38 (31–69)/95	36 (30.6–46)/67	71.6 (35.5–455)/28	0.001
>60, *n* (%)	26 (27)/95	10 (15)/67	16 (57)/28	<0.001
Creatine kinase-MB (U/L), median(IQR)^#^	36.4 (28–53.5)/98	34 (25.7–45.9)/69	45.9 (34.9–88)/29	0.002
>40, *n* (%)	38 (39)/98	22 (32)/69	16 (55)/29	0.031
Lactate dehydrogenase (U/L), median(IQR)^#^	427 (343–540)/99	403 (317–503)/70	527 (418–1560)/29	<0.001
>322, *n* (%)	78 (79)/99	51 (73)/70	27 (93)/29	0.025
Hemoglobin (g/L), median(IQR)**^#^**	152 (138–175)/102	155 (142–176)/73	136 (120–175)/29	0.034
Prothrombin time (s), median(IQR)**^#^**	13 (12–15.5)/57	12 (12–13)/33	15.8 (13.3–23.5)/24	<0.001
Activated partial thromboplastin time (s), median(IQR)**^#^**	48.6 (45–53)/57	48 (43–49)/33	54.8 (46.8-71)/24	0.001
D-dimer (mg/L), median(IQR)**^#^**	1.3 (0.8–4.6)/45	1.1 (0.8–1.7)/25	2.4 (1.1–17.1)/20	0.020
Albumin (g/L), median(IQR)**^#^**	33.6 (31–35.2)/86	34 (32–36)/59	31 (26–34.2)/27	0.001
Total bilirubin (mmol/L), median(IQR)**^#^**	89.5 (66.2–134.5)/100	90 (68–136)/71	86(54.5–133)/29	0.779
Direct bilirubin (mmol/L), median(IQR)**^#^**	9.6(8–12)/99	9(7–12)/70	10.5(9–14.2)/29	0.002

IQR, interquartile range; TEN, total enteral nutrition; PPN, partial parenteral nutrition; TPN, total parenteral nutrition; The variables with fixed reference ranges were transformed into categorical ones in the regression analysis, while those with different reference ranges in neonates of different gestational ages were still analysed as continuous variables.

^#^ There were missing value and the number after the slash is the number of non-missing value.

^a^ *P* for Pearson's Chi-squared tests with Yates’ continuity correction, otherwise, *P* for Pearson's Chi-squared tests or Wilcoxon rank sum tests.

### Clinical manifestations

In this cohort, the most common initial symptoms of E-11 infection were fever (52%) and tachypnoea (21%). During the course of illness, haemorrhage (including petechiae, gastric bleeding or bloody stools) was the most common clinical symptoms (46%). The most common complications included acute myocardial injury (41%, as evidenced by elevated myocardial enzyme levels and decreased cardiac function on echocardiography), pneumonia (22%), acute respiratory distress syndrome (ARDS; 16%, excluding RDS owing to surfactant deficiency), DIC (10%), acute renal injury (9%, with elevated creatinine levels with or without oliguria/anuria) and shock (9%). However, in the severe group, all patients presented with hepatic dysfunction and coagulopathy (*P* < 0.001) (Supplementary Table S1).

### Laboratory findings

**Figure 1:**
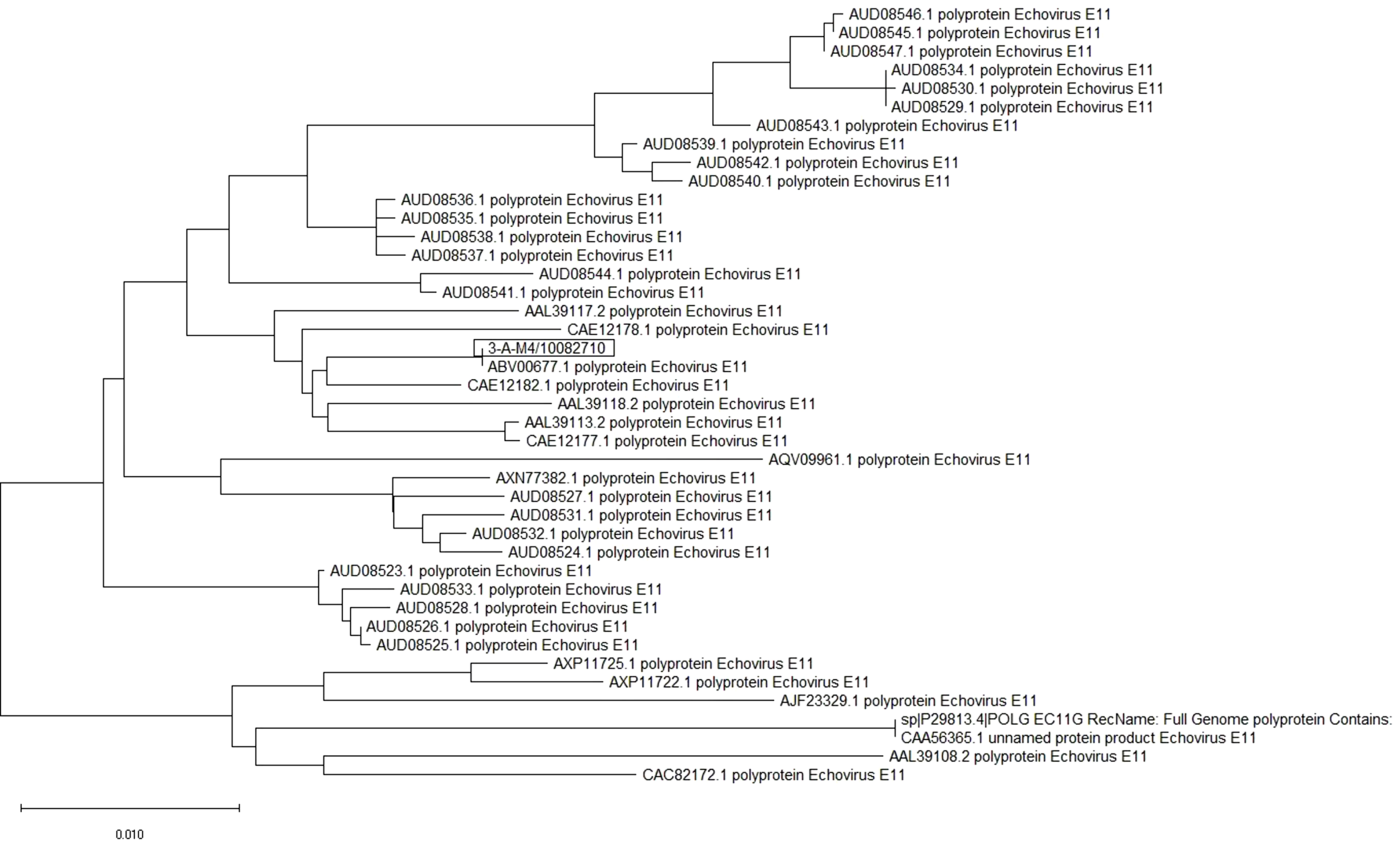
E-11 sequence information and phylogenetic analysis. Sequence of E-11 isolated from two patients in Guangzhou Women and Children's Medical Center was shown. It revealed that GWCMC01/GZ/CHN/2019 is 99% consistent with E-11 strain D207 (GenBank No. EF634316).

The whole-genome RNA of E-11 from patients in Guangzhou was examined. We named the virus as E-11 strain GWCMC01/GZ/CHN/2019, whose sequence information was 99% consistent with the E-11 strain D207 (GenBank No. EF634316) that was isolated in Slovakia in 2007 (Figure 1).

Within the first three days of the disease onset, white blood cell (WBC) counts were mostly within the normal range. Abnormalities seen within the first few days of the disease onset included decreased platelet (PLT; 16%) and lymphocyte counts (10%) and increased serum creatine kinase-MB (CK–MB) levels (39%), which were substantially elevated (50%, 14% and 55%, respectively) in the severe group from the beginning of disease onset (Table 1). Thrombocytopenia (PLT < 140 × 10⁹/L) was rare in the mild group(1%). However, half of patients in the severe group (50%) had an early onset of thrombocytopenia within the first three days (*P *< 0.001) (Table 1).

### Treatment and outcomes

Most patients in the mild group only required routine care. In severe cases, transfusion (60%) was the most common treatment, followed by mechanical ventilation (57%), vasopressors (53%) and intravenous immunoglobulin (47%). In addition, some patients in the severe group were critically ill and received extensive multi-organ/system support including steroids (27%), plasmapheresis (10%) and/or continuous renal replacement therapy (CRRT; 7%). There was no death in the mild group. The median duration of hospitalisation in mild and severe groups were 7 (6–9) and 14 (8–21) days, respectively.

Patients who survived were followed up after discharge until approximately 4 months of age. All patients in the mild group remained healthy with normal development. However, there were six death and two cases of prolonged hepatic dysfunction after haemorrhage-hepatitis syndrome in the severe group (Supplementary Table S1).

### Risk factors and early markers of haemorrhage-hepatitis syndrome

Risk factors and early markers associated with haemorrhage-hepatitis syndrome and poor outcomes are demonstrated in Table 2. Infants born prematurely, had premature rupture of foetal membrane (PROM), receiving PN (TPN) at the disease onset had increased risk of developing haemorrhage-hepatitis syndrome, while age at the disease onset ≥8 days may be a protective factor. PLT < 140 × 10⁹/L within the first three days of onset, alanine transaminase (ALT) > 50 U/L and aspartate transaminase (AST) > 60 U/L in the first week of disease onset, lactic dehydrogenase (LDH) > 322 U/L and CK-MB > 40 U/L were associated with haemorrhage-hepatitis syndrome. We also found that the degree of change in several factors was associated with the severity of the disease, which include decreased haemoglobin levels (per 10 g/L; odds ratio [OR], 1.2; 95% confidence interval [CI], 1.0–1.4), increased PT (OR, 1.9; 95% CI, 1.3–2.8), activated partial thromboplastin time (APTT) (OR, 1.1; 95% CI, 1–1.2), D-dimer (OR, 1.2; 95% CI, 1–1.3), and direct bilirubin levels (OR, 1.2; 95% CI, 1–1.3).

**Table 2:** Univariate and multivariate logistic regression analysis of risk factors/early markers for severe E-11 infection or death/prolonged liver dysfunction.

Risk Factors/early markers	Outcome = Severe				Outcome = Death/prolonged liver dysfunction			
	Complete case analysis^†^		Multiple imputation analysis		Complete case analysis^†^		Multiple imputation analysis	
	Univariate analysis, OR (95% CI)	Multivariate analysis, (95% CI)	Univariate, OR (95% CI)	Multivariate analysis, (95% CI)	Univariate analysis, OR (95% CI)	Multivariate analysis, OR(95% CI)	Univariate analysis, OR (95% CI)	Multivariate analysis, (95% CI)
Risk factors Gestational age (week), ref: ≥ 37	7.3 (2.7–19.9)^*^		7.3 (2.7–19.9)^*^		13.2 (2.5–70.7)*		13.2 (2.5–70.7)*	9.9 (0.2–516.2)
Birthweight (g), ref:≥2500	4.9 (1.6–15.5)*		4.9 (1.6–15.5)*		7.8 (1.7–35.8)*		7.8 (1.7–35.8)*	
Age of disease onset, ref:<3d								
3–7d	1.6 (0.5–5.6)		1.6 (0.5–5.6)		4.0 (0.6–25.0)		4.0 (0.6–25.0)	
≥8d	0.4 (0.1–1.0)*		0.4 (0.1–1.0)*		0.4 (0.0–3.0)		0.4 (0.0–3.0)	
**Feeding pattern at onset, ref:TEN**								
PPN	5.9 (2–17.2)*		5.9 (2–17.2)*	12.9 (2.2–77.5)*	1.6 (0.1–18.5)			
TPN	10.6 (2.9–38.3)*		10.6 (2.9–38.3)*	28.7 (2.8–295.1)*	18.6 (3.1–110.5)*			
Premature rupture of membrane, ref:no	3.5 (1.1–11.5)*		3.5 (1.1–11.5)*		5.2 (1.1–25.2)*		5.2 (1.1–25.2)*	
Early markers Platelet count (×10⁹/L), ref:≥140	72.0 (8.8–587.5)^*^^#^	47.7 (5.2–441.1)*	33.6 (6.8–164.5)^*^	17.7 (1.4–221.5)*	25.5 (4.5–143.7)^*^		23.0 (4.1–128.4)^*^	1.2 (0.0–335.2)
Alanine aminotransferase (U/L), ref:≤50	42.1 (8.1−+∞)^*^^#^		41.9 (8.1−+∞)^*^^#^	+∞(0.0−+∞)	37.1 (6.5–212.8)^*^		29.3 (4.7–182.1)^*^	+∞(0.1−+∞)
Aspartate aminotransferase (U/L), ref:≤60	7.6 (2.8–20.8)^*^		5.8 (2.3–14.6)^*^		10.1 (1.9–53.7)*		7.8 (1.5–41.5)*	
Creatine kinase-MB (U/L), ref:≤40	2.6 (1.1–6.4)*		2.5 (1.0–6.1)*		2.9 (0.6–12.8)		2.6 (0.6–11.7)	
Lactate dehydrogenase (U/L), ref:≤322	5.0 (1.1–23.2)^*^		5.4 (1.2–24.8)*		3.2 (0.6−+∞)^#^		3.3 (0.6−+∞)^#^	
Hemoglobin, per 10 g/L decrease	1.2 (1.0–1.4)*		1.2 (1.1–1.4)*	1.5 (1.1–2.0)*	1.3 (1.1–1.6)*		1.3 (1.4–3.4)*	
Prothrombin time, s	1.9 (1.3–2.8)*		1.1 (1.0–1.1)		1.6 (1.1–2.3)*	1.5 (1.0–2.3)*	1.1 (1.0–1.1)*	1.0 (0.9–1.1)
Activated partial thromboplastin time, s	1.1 (1–1.2)*		1.1 (1.0–1.1)*		1.1 (1.0–1.2)*		1.1 (1.0–1.1)*	
D-dimer, mg/L	1.2 (1–1.3)*		1.0 (1.0–1.0)		1.1 (1.0–1.2)*		1.0 (1.0–1.1)	
Albumin, g/L	0.8 (0.7–0.9)*		0.9 (0.8–1.0)*	0.9 (0.8–1.0)	0.9 (0.8–1.0)*		0.9 (0.8–1.0)*	0.7 (0.4–1.3)
Direct bilirubin, mmol/L	1.2 (1.0–1.3)*		1.2 (1.0–1.3)*	1.1 (0.9–1.3)	1.2 (1.0–1.3)*		1.2 (1.0–1.3)*	

TEN, total enteral nutrition; PPN, partial parenteral nutrition; TPN, total parenteral nutrition; OR, odds ratio; CI, confidence interval.

^#^ Exact test.

*P < 0.05.

**P < 0.001.

^†^ The number of cases in both complete case analysis was 42 for multivariate analysis.

In the multiple imputation analysis, the results of the univariate analysis were similar to those of complete case analysis. According to the aggregation model, TPN (OR, 28.7; 95% CI, 2.8–295.1) and PPN (OR, 12.9; 95% CI, 2.2–77.5) at the onset of disease, decreased haemoglobin levels (per 10 g/L; OR, 1.5; 95% CI, 1.1–2.0) and PLT <140 × 10⁹/L (OR, 17.7; 95% CI, 1.4–221.5) were associated with haemorrhage-hepatitis syndrome. However, for the outcome of death/prolonged liver dysfunction, there were no statistical significance in multivariate regression for any variables (Table 2).

### Immunological testing

**Figure 2:**
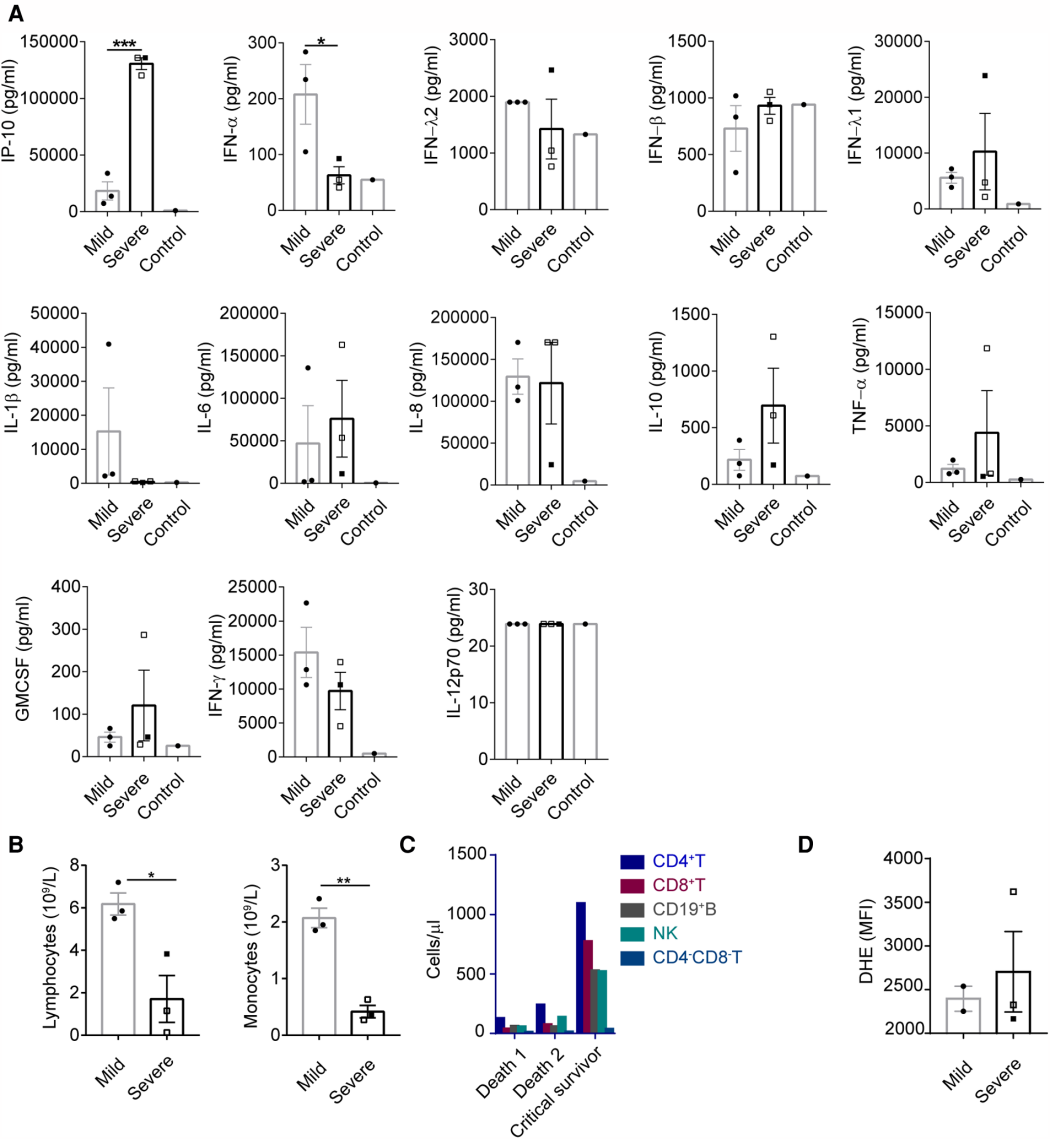
Impaired anti-viral capacity and increased inflammation were observed in patients with haemorrhage-hepatitis syndrome associated with severe E-11 infection. (**A**) Concentrations of maximum cytokine levels of plasma from E-11 infected subjects during hospitalization (severe, *n* = 3; mild, *n* = 3) and healthy control (*n* = 1). Cytokine levels were determined using LEGENDplex™ Human Anti-Virus Response Panel (13-plex). (**A–C**) Empty square represented patients that had passed away. *P* values were calculated by unpaired t test. *, *P* < 0.05; **, *P* < 0.01; ***, *P* < 0.0005; ****, *P* < 0.0001. (**B**) Lymphocyte and monocyte count from blood of E-11 infected subjects between 7 and 10 days during the course of infection (severe, *n* = 3; mild, *n* = 3). (**C**) CD4 + T, CD8 + T, CD19 + B, NK and CD4-CD8-T cell count from blood of E-11 infected subjects in the 10th day in the course of infection. (**D**) Peripheral blood samples from E-11 infected subjects post-diagnosis (severe, *n* = 3; mild, *n* = 2) were stimulated with Phorbol-12-myristate-13-acetate (PMA). Neutrophil ROS production was measured by flow cytometry using the Dihydroethidium, DHE dye. PMA induced neutrophil ROS production measured by the DHR MFI in were shown as the median (IQR).

There were six patients with nosocomial E-11 infection from the same ward in Guangdong province. Of the six patients, four had severe disease and two had mild infection. The levels of proinflammatory cytokines (IP-10, IL-1β, IL-6, IL-8, TNF-α and GM-CSF) and anti-virus-related factors (IFN-α, IFN-*λ*1 and IFN-*γ*) were upregulated in the blood samples of the six patients as compared those of the control patients without infection. Patients with haemorrhage-hepatitis syndrome had lower concentrations of IFN-α, IFN-*λ*2/3, IL-1β and IFN-*γ* and higher levels of IP-10, IFN-*λ*1, IL-6, IL-10, TNF-α and GM-CSF. Among these, the difference in the protein levels of IFN-α (*P *< 0.05) and IP-10 (*P *< 0.0005) reached statistical significance (Figure 2A).

As demonstrated in Figure 2B, the absolute lymphocyte and monocyte counts were significantly lower in the severe group than those in the mild group. The subsets of CD3+, CD4+, CD8+, CD19 + and natural killer (NK) cells were higher in patients with severe disease who survived than in those who died (Figure 2C). No difference was observed in the ability of neutrophils to generate reactive oxygen species (ROS) between the groups (Figure 2D).

## Discussion

Despite being an important cause of haemorrhage-hepatitis syndrome or death, E-11 infection in neonates has not been sufficiently studied (18–20). With a larger sample size than previous studies, our study compared the characteristics of patients with and without E-11 associated haemorrhage-hepatitis syndrome.

Data from previous studies suggest that more than 90% of patients with E-11 infection are asymptomatic or present with mild fever (2). However, in our cohort of neonates, we found that 29% of the infected neonates were severe cases and the case-fatality rate was 6%. Consistent with some other studies, severe E-11 infection in our neonates was associated with haemorrhage-hepatitis syndrome that causes hepatitis with liver dysfunction and coagulopathy (4, 5, 7, 8, 20). Also different from previous reports, respiratory symptoms were more common initial symptoms than gastrointestinal symptoms, and aseptic meningitis was not commonly identified in our study (3). Although generally mild, acute myocardial injury was the most common complication in our cohort. These differences may be related to difference in the age groups of the study population, and subgroups of enterovirus. In addition, a previous study from Li et al. has reported that there have been multiple genotypes of E-11, with different pathogenicity and clinical features, circulated in mainland China (21). Although the E-11genotype identified in our study is most closely related to E-11 strain D207, whether these clinical features found in our cohort are characteristic to this E-11 genotype need to be further studied.

Mortality was high (20%) in the severe group, who had variable degrees of hepatic dysfunction in our study. Early identification of neonates with the trend of haemorrhage-hepatitis syndrome is therefore crucial for prompt treatment. Previous studies have reported that factors associated with hepatic necrosis with coagulopathy in echovirus infection include prematurity, maternal history of illness, early age of onset, higher WBC count and lower haemoglobin levels (21, 22). Similarly, we found that prematurity, early age of onset (<3 days) and lower haemoglobin levels were significantly more common in severe cases. In addition, PN at disease onset is associated with an increased risk of haemorrhage-hepatitis syndrome. Moreover, an early decrease in PLT by <140 × 10⁹/L within the first three days of illness is an early marker of severe disease. These risk factors and early markers could be helpful for early identification of haemorrhage-hepatitis syndrome. Multiple imputation may reduce bias and increase power compared with complete case analysis (23). Gestational age and other variables appeared only in multiple imputation analysis, possibly due to the inclusion of larger numbers of study subjects in multiple imputation analysis, allowing more variables to achieve statistical significance.

Some studies have indicated that passive transplacental acquisition of antibodies prevents severe, systemic echovirus disease or may be the reason for asymptomatic infection (7, 24). However, the immune characteristics of haemorrhage-hepatitis syndrome remain unclear owing to limited studies. Previous studies have demonstrated that E-11 infection affects specific cell populations in the human intestine (25). In our study, we detected elevated IP-10 and decreased IFN-α in critically ill neonates. Meanwhile we also found elevated IP-10 level in the livers of patients with haemorrhage-hepatitis syndrome (data not shown). IP-10 has been shown to correlate with hepatic injury (26–30). However, whether this can be used as a marker of disease progression associated with haemorrhage-hepatitis syndrome in neonates needs to be further studied.

IP-10 could have been induced to recruit more lymphocytes as a feedback to low levels of IFN-α in cases with haemorrhage-hepatitis syndrome. Lin GL reported decreased production of type 1 IFNs as a feature of the neonatal immune system (31). We identified reduced IFN-α levels in neonates with severe disease as compared with the other neonatal patients. IFN-α is secreted by immune cells and epithelial cells and has an anti-enterovirus effect, which may restrict enterovirus replication in the human intestine (32). It has been shown that the cellular sources of IFN-α may vary during different viral infections and epithelial cells in the gut produce IFN-I in response to mucosal infections caused by different viruses (33, 34). We speculate that incomplete colonisation of intestinal flora resulting from inadequate enteral nutrition may have contributed to either immature immune function or dysfunction of intestinal epithelial cells, and thus resulting in the downregulation of IFN-α in infants with severe disease in our cohort. A study showed that some elemental diet can regulate the immunological response of intestinal tract (35). Given that PN is a risk factor for haemorrhage-hepatitis syndrome in our study and previous studies (36), we speculate that lack of enteral nutrition may aggravate deficiency of gut immune function, which plays an important role in adaptive immune insufficiency and further lead to haemorrhage-hepatitis syndrome in some neonatal patients.

Our study has some notable limitations. First, there were variations in medical treatment among different centres, and laboratory testing were not completely performed in a few centres. Second, given the retrospective nature of the study, maternal E-11 testing was not performed. Third, the patients were followed up until 4 months of age; therefore, we could not report long-term outcome data, especially long-term neurodevelopmental outcomes (37). Lastly, only one center conducted immunological testing in patients; therefore, the immunological changes found in our study needs to be confirmed by further studies. However, the new risk factors and early markers found in our study may aid the early identification of infants with haemorrhage-hepatitis syndrome that enable early intervention.

In conclusion, we found that PN at onset, thrombocytopenia and decreased haemoglobin levels in the early stage of illness are associated with haemorrhage-hepatitis syndrome in neonates with E-11 infection. We speculate that intestinal immune insufficiency may play a key role in the development of haemorrhage-hepatitis syndrome in neonates, which should be verified by future studies.

## Data Availability

The datasets presented in this study can be found in online repositories. The names of the repository/repositories and accession number(s) can be found in the article/**Supplementary Material**.
