# Supplementary material for: Risk factors and early markers for echovirus type 11 associated haemorrhage-hepatitis syndrome in neonates, a retrospective cohort study
Source: Front Pediatr. 2023 Apr 6;11:1063558. doi: 10.3389/fped.2023.1063558 (PMC10117901; doi:10.3389/fped.2023.1063558)
Supplement: Supplementary file 1 [file Table1.docx]

**Supplementary Material**

**eTable 1. Supplement of Clinical Characteristics of the study population**

|  | **All Patients**  (n=105) | **Mild**  (n=75) | **Severe**  (n=30) | ***P* value** |
| --- | --- | --- | --- | --- |
| **Initial symptoms/signs** |  |  |  |  |
| Fever, n(%) | 55(52) | 45(60) | 10(33) | 0.013 |
| Vomiting, n(%) | 4(4) | 0(0) | 4(13) | 0.008^a^ |
| Tachypnea, n(%) | 22(21) | 13(17) | 9(30) | 0.150 |
| Hemorrhage, n(%) | 4(4) | 2(3) | 2(7) | 0.687^a^ |
| Others, n(%) | 20(19) | 15(20) | 5(17) | 0.694 |
| **Subsequent clinical symptoms/signs** |  |  |  |  |
| Respiratory distress, n(%) | 15(14) | 1(1) | 14(47) | <0.001 |
| Jaundice, n(%) | 21(20) | 5(7) | 16(53) | <0.001 |
| Hemorrhage, n(%) | 48(46) | 30(40) | 18(60) | 0.063 |
| Oliguria or nasarca, n(%) | 7(7) | 2(3) | 5(17) | 0.030^a^ |
| Lethargy or seizures, n(%) | 15(14) | 6(8) | 9(30) | 0.004 |
| **Laboratory findings** |  |  |  |  |
| Potassium (mmol/L), median(IQR)^#^ | 4.5(4.1-4.8)/98 | 4.5(4.1-4.8)/69 | 4.3(3.9-4.9)/29 | 0.567 |
| Sodium (mmol/L), median(IQR)^#^ | 136.7(135-139.7)/98 | 137(135-139)/69 | 136.7(134-140)/29 | 0.770 |
| Creatinine (μmol/L), median(IQR)^#^ | 28.5(22-36.5)/96 | 27.3(22-34)/69 | 34.1(19.7-55)/27 | 0.218 |
| ≤97, n(%) | 95(99)/96 | 69(100)/69 | 26(96)/27 | 0.281^b^ |
| >97, n(%) | 1(1)/96 | 0(0)/69 | 1(4)/27 |  |
| Urea nitrogen (μmol/L), median(IQR)^#^ | 3.1(2.3-3.9)/92 | 3.2(2.4-3.8)/69 | 2.9(1.9-4.9)/24 | 0.666 |
| Creatine kinase (U/L), median(IQR)^#^ | 123.5(79-223)/98 | 118(77-204)/68 | 168(102-388.7)/29 | 0.064 |
| ≤390, n(%) | 84(86)/98 | 62(90) | 22(76) | 0.071 |
| >390, n(%) | 14(14)/98 | 7(10) | 7(24) |  |
| C-reactive protein(mg/L) |  |  |  |  |
| <6, n(%) | 67(68)/99 | 46(66)/70 | 21(72)/29 | 0.807 |
| ≥6, n(%) | 32(32)/99 | 24(34)/70 | 8(28)/29 |  |
| **Complications,** **n(%)** |  |  |  |  |
| Pneumonia | 23(22) | 4(5) | 19(63) | <0.001 |
| Acute respiratory distress syndrome | 17(16) | 0(0) | 17(57) | <0.001 |
| Acute myocardial injury | 43(41) | 23(31) | 20(67) | 0.001 |
| Acute renal injury | 9(9) | 0(0) | 9(30) | <0.001^a^ |
| Hepatic dysfunction and coagulation | 30(29) | 0(0) | 30(100) | <0.001^a^ |
| Meningitis | 18(17) | 11(15) | 7(23) | 0.287 |
| [Disseminated intravascular coagulation](http://dict.youdao.com/w/disseminated%20intravascular%20coagulation/#keyfrom=E2Ctranslation) | 10(10) | 0(0) | 10(33) | <0.001^a^ |
| Shock | 9(9) | 0(0) | 9(30) | <0.001^a^ |
| **Treatments, n(%)** |  |  |  |  |
| CPAP/CMV/HFOV | 17(16) | 0(0) | 17(57) | <0.001 |
| Fluid resuscitation | 11(10) | 0(0) | 11(37) | <0.001^a^ |
| Vasopressors | 17(16) | 1(1) | 16(53) | <0.001 |
| Transfusion(RBC/FFP/PLT) | 21(20) | 3(4) | 18(60) | <0.001 |
| IVIG | 20(19) | 6(8) | 14(47) | <0.001 |
| Steroid | 8(8) | 0(0) | 8(27) | <0.001^a^ |
| Plasmapheresis | 3(3) | 0(0) | 3(10) | 0.033^a^ |
| CRRT | 2(2) | 0(0) | 2(7) | 0.142^a^ |
| **Pathogen test** |  |  |  |  |
| Specimens positive for E-11, n(%) |  |  |  |  |
| Serum | 47(45) | 31(41) | 16(53) | 0.264 |
| Rectal swabs | 61(58) | 46(64) | 15(50) | 0.192 |
| Throat swabs | 6(6) | 1(1) | 5(17) | 0.012^a^ |
| Cerebrospinal fluid | 10(10) | 4(6) | 6(20) | 0.062^a^ |
| **Outcome** |  |  |  |  |
| Duration of stay (day), median(IQR) | 8(6-13) | 7(6-9) | 14(8-21) | <0.001 |
| Discharge outcome, n(%) |  |  |  |  |
| Without sequela | 97(92) | 75(100) | 22(73) | <0.001 |
| Death | 6(6) | 0(0) | 6(20) |  |
| Liver dysfunction at 4 month | 2(2) | 0(0) | 2(7) |  |

Abbreviation: IQR = interquartile range, CPAP=continuous positive airway pressure, CMV=controlled mechanical ventilation, HFO=high frequency oscillatory ventilation, RBC=red blood cell, FFP=fresh frozen plasma, PLT=platelet, IVIG=intravenous immunoglobulin, CRRT= renal replacement therapy, E-11=echovirus type 11. ^a^*P* for Pearson’s Chi-squared tests with Yates’ continuity correction, ^b^*P* for Fisher’s exact tests, otherwise, *P* for Pearson’s Chi-squared tests or Wilcoxon rank sum tests. ^#^There were missing value and the number after the slash is the number of non-missing value.
